# Plant responses to abiotic stress regulated by histone acetylation

**DOI:** 10.3389/fpls.2024.1404977

**Published:** 2024-07-16

**Authors:** Fei Wang, Chong-Hua Li, Ying Liu, Ling-Feng He, Ping Li, Jun-Xin Guo, Na Zhang, Bing Zhao, Yang-Dong Guo

**Affiliations:** ^1^ College of Horticulture, China Agricultural University, Beijing, China; ^2^ Sanya Institute of China Agricultural University, Sanya, China

**Keywords:** abiotic stress, epigenetic regulation, histone acetylation, histone acetyltransferase, histone deacetylase

## Abstract

In eukaryotes, histone acetylation and deacetylation play an important role in the regulation of gene expression. Histone acetylation levels are reversibly regulated by histone acetyltransferases (HATs) and histone deacetylases (HDACs). Increasing evidence highlights histone acetylation plays essential roles in the regulation of gene expression in plant response to environmental stress. In this review, we discussed the recent advance of histone acetylation in the regulation of abiotic stress responses including temperature, light, salt and drought stress. This information will contribute to our understanding of how plants adapt to environmental changes. As the mechanisms of epigenetic regulation are conserved in many plants, research in this field has potential applications in improvement of agricultural productivity.

## Introduction

As sessile organism, the growth and development of plants are constantly affected by environmental conditions. Adverse environmental conditions severely affect the growth and productivity of crop plants. Abiotic stress disrupts the growth and development of crop plants, leading to the reduction of quality and yield, which is one of the main factors restricting the yield of crop plants in China (Upadhyay et al., 2019; Salava et al., 2021). Many studies have been made to reveal the mechanism of plants response to stress conditions.

Epigenetics explores heritable alterations in gene expression without changes to DNA sequence itself (Stam, 2009; Gao et al., 2021b). This field examines a variety of phenomena, including DNA methylation, genomic imprinting, gene silencing, RNA editing, defense against transposon proliferation, etc (He et al., 2011; Raissig et al., 2011; Nuñez et al., 2021). Additionally, in plants, epigenetic mechanisms play crucial roles in development and in responding to environmental stressors (Rando and Chang, 2012; Berry and Dean, 2015). In eukaryotic cells, DNA wraps around core histone proteins -H2A, H2B, H3, and H4 to form chromatin (Du et al., 2020). These histone proteins undergo various post-translational modifications including acetylation, methylation, phosphorylation, ubiquitination, SUMOylation, and ADP ribosylation, impacting gene expression and activity (Ueda and Seki, 2020).

**Figure 1:**
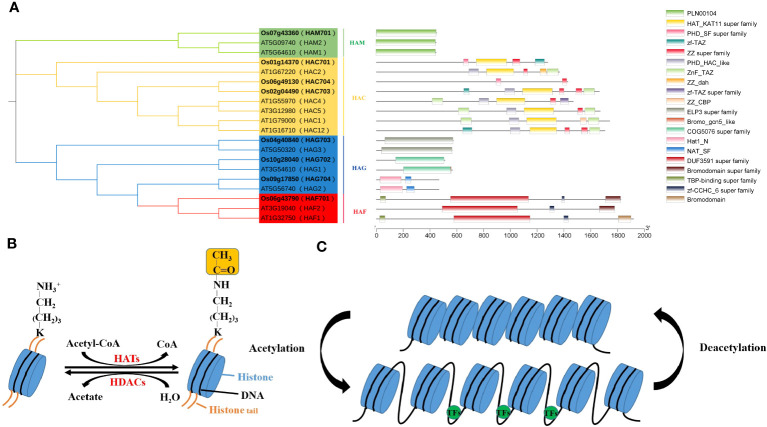
Conserved domain analysis of the AtHAT/OsHAT gene families **(A)** and regulation of histone acetylation dynamics **(B, C)**.

Histone acetylation is a crucial epigenetic modification, regulates gene expression in eukaryotes (Gan et al., 2021; Xu et al., 2022), affecting plant growth, development, and stress response (**Supplementary Table 2**). Histone acetylation levels are reversibly regulated by histone acetyltransferases (HATs) and histone deacetylases (HDACs) (Jiang et al., 2020; Zheng et al., 2020) (**Figure 1B**). HATs add acetyl groups to specific lysine residues on N-termini of histone H3 and H4, which activates gene transcription (**Figures 1B, C**). This process neutralizes the positive charge on the histone tail, reducing the interaction between histone and DNA or other histones, thereby loosening chromatin structure. This allows transcription factors (TFs) easier access to target genes, facilitating the regulation of downstream gene expression. On the contrary, HDACs are associated to transcriptional repression and gene silencing by removing acetyl groups from lysine residues (de Rooij et al., 2020) (**Figure 1C**).

Histone acetylation typically regulates by recruiting TFs to modulate acetylation levels at various downstream gene promoter sites. It primarily occurs on conserved lysine residues at the N terminal of H3 and H4, with modification sites include H3 (K4, K9, K14, K18, K23, K27) and H4 (K5, K8, K12, K16). In plants, HATs are categorized into four families based on domain characteristics: GNAT (Gcn5-related N-acetyltransferases), MYST (MOZ-YBF2/SAS3-SAS2/TIP60), CBP (CREB-binding protein) and TAFII250 (TATA-binding protein-associated factor), also known as HAG, HAM, HAC, and HAF, respectively (**Figure 1A**). These HAT families possess distinct conserved domains granting them multifunctional capabilities (**Supplementary Table 1**). For example, HAG ELP3 (elongator complex protein 3) interacts with RNA Pol II, HAT1 (histone acetyltransferase 1) conducts histone acetylation, Znf-ZZ and Znf-TAZ facilitates protein-protein interactions, and the PHD domain enables HATs to interact with other histones. Thus, HATs form protein complexes to collaboratively regulate gene expression with TFs and other histone modifiers.

The number of HATs varies among plant species, with *Arabidopsis* having 12, rice 8, and tomato 32. Research indicates that histone acetylation plays a crucial role in plant responses to various stresses, including light (Perrella et al., 2020; Ageeva-Kieferle et al., 2021), temperature (Hu et al., 2015; Ohama et al., 2017), salt (Zheng et al., 2021; Feng et al., 2022) and ABA (Chen et al., 2010; Liao et al., 2016).

Accordingly, it is critically important to conduct research on the epigenetic regulation of crop plants under abiotic stress. This review encapsulates the advancements in understanding histone acetylation in plant responses to abiotic stress, providing key epigenetic insights for the genetic enhancement of future crops.

## Functions of histone acetylation in plants response to abiotic stress

### Salt stress

HATs/HDACs are pivotal in managing salt stress by regulating the expression of salt stress responsive genes. In *Arabidopsis*, histone acetyltransferase GCN5 regulated the expression of cellulose synthesis genes to maintain cell wall integrity by altering the acetylation level at H3K9 and H3K14, thereby improving salt stress tolerance (Zheng et al., 2019). In contrast, more histone deacetylases are involved in salt stress regulation. For example, HD2C interacted with another histone deacetylase HDA6, regulating ABA-responsive genes expression by changing histone H3K9 and H3K14 acetylation, responding to ABA and salt stress (Luo et al., 2012). Overexpression of the *HDA15* gene conferred resistance to salt stress by regulating the H3K14ac and H4K16ac levels of *NCED3* (Ueda et al., 2017; Truong et al., 2021). Research indicates that WRKY53 and HDA9 play contrasting roles in regulating plant response to salt stress (Zheng et al., 2020). Moreover, HY5 was found to collaborate with HDA9 to regulate the transcription of *HsfA2* in response to salt stress (Yang et al., 2023b). Histone deacetylase AtSRT2 was shown to regulate salt tolerance during seed germination via repression of vesicle-associated membrane protein 714 (*VAMP714*) (Tang et al., 2022). The Histone Deacetylase Complex 1 (HDC1) modulated the response of salt-treated seedlings by changing the acetylation levels at histone H3 lysine 9 and 14 (H3K9ac/H3K14ac) (Perrella et al., 2023). SAP18 interacted with HDA1, exerting a negative regulatory effect on the adaption to salt stress (Song and Galbraith, 2006). Furthermore, MSI1, HDA19 and HDC1complex was identified to interact with SIN3-like proteins, collectively contributing to the intricate regulatory network that governs salt stress tolerance (Mehdi et al., 2016).

In rice, *OsHAC701* has been reported to respond to salt treatment (Liu et al., 2012). OsHDA706, a histone H4 deacetylase, could enhance the salt tolerance of rice by regulating the expression of *OsPP2C49* via H4K5 and H4K8 deacetylation (Liu et al., 2023). IDS1 interacted with histone deacetylase HDA1 to regulate rice salt tolerance by repressing the expression of *LEA1* and *SOS1* (Cheng et al., 2018). Another histone deacetylase, HDA710, controlled salt tolerance by regulating H4K5 and H4K16 acetylation on genes responsive to ABA (Ullah et al., 2020). HDA704 directly bound to *DST* and *ABIL2*, repressing their expression to positively regulate drought and salt tolerance (Zhao et al., 2021).

HATs/HDACs are also involved in salt stress regulation in other plants such as wheat, maize, soybean, cotton, poplar. In wheat, TaHAG1, a histone acetyltransferase, enhancing salt tolerance by modifying H3K9ac and/or H3K14ac at TSSs (Transcription Start Sites) (Zheng et al., 2021). Maize’s *ZmHATB* and *ZmGCN5* boost salt tolerance by elevating H3K9ac levels at *ZmEXPB2* and *ZmXET1* promoters, causing root swelling (Li et al., 2014). GmPHD5 interacted with acetyltransferase GmGNAT to regulate salt responsive genes in soybean through H3K14ac (Wu et al., 2011). A transcription factor GsNAC83 in wild soybean, interacts with histone acetyltransferase GsMYST1 and GsSnRK1 kinase, to increase *COR15B* promoter activity for better tolerance to salt stress (Feng et al., 2022). Under salt stress, GmNFYA likely accumulated and competed with GmHDA13 for interaction with GmFVE, reducing H3K9ac at target loci and improving tolerance in soybean (Lu et al., 2021). Cotton expresses genes like *GhHAC1501* and *GhHAG1504* expressed higher under salt stress (Imran et al., 2019). Histone deacetylase gene *PtHDT902* negatively regulated salt stress tolerance in poplar (Ma et al., 2020). While expression changes in *SiHAT17*, *SiHAT23* and *SbHDACs* under salt stress indicated their role in stress management (Du et al., 2022; Xing et al., 2022).

### Drought stress

Drought stress is one of the important abiotic stresses, which can cause serious harm to plants. Histone acetylation is widely involved in managing this stress. ABA-Responsive Element Binding Protein 1 (AREB1) and the ADA2b-GCN5 HAT complex regulate the expression of the drought-responsive genes (*PtrNAC006*, *PtrNAC007* and *PtrNAC120*) by enhancing H3K9ac under drought stress conditions, suggesting that transcription factors coordinated with histone acetylation to play important role in response to drought stress (Li et al., 2019). CRISPR/dCas9 -AtHAC1 fusion improves drought tolerance in *Arabidopsis* by activating *AREB1* and *RD29A* (Roca Paixão et al., 2019). Rice, wheat and Chinese cabbage show increased expression of various HAT genes under drought conditions, indicating their involvement in drought response (Fang et al., 2014; Eom and Hyun, 2018; Tan et al., 2019; Hou et al., 2021; Li et al., 2022).

GhHDT4D may enhance drought tolerance by suppressing *GhWRKY33* via reducing its H3K9ac, thereby activating the downstream drought response genes in cotton (Zhang et al., 2020a). *SlHDA1* and *SlHDA3* (Guo et al., 2022a; Guo and Wang, 2023a), and the interaction between HD2A and HD2C functions by H3K9ac in stomatal closure and root growth (Tahir et al., 2022), underscore HAT/HDAC’s role in drought stress management. HDA9 interacted with PWR-ABI4 complex to promote drought tolerance, contrasting with WRKY53’s effect under drought stress (Ali and Yun, 2020; Zheng et al., 2020). HDT4 worked with ENAP1-ENAP2-MYB44 complex to regulate drought responsive genes by altering H3K27ac (Zhao et al., 2022a). A histone deacetylase of *Brachypodium distachyon*, BdHD1 regulated the expression of *BdWRKY24* by changing H3K9ac to positively regulate drought response (Song et al., 2019b). Meanwhile, BdHD1 interacted with two drought-responsive transcription factors, BdWRKY24 and BdMYB22 to combat drought stress (Song et al., 2020). In conclusion, HAT/HDAC typically engages with ABA-related transcription factors such as AREB and WRKY members to participate in drought-related gene regulation. However, more regulatory elements, including additional transcription factors requires identification.

### Temperature stress

Temperature stress, both high and low, hinders growth and severely affects their life processes. Plants have evolved mechanisms to adapt and attenuate the hazards of temperature stress, with HAT/HDACs playing a significant role. Heat stress increases the expression of HAT genes in rose, suggesting histone acetylation adjustments in response (Wu et al., 2022). GCN5 regulated heat stress responsive genes *HSFA3* and *UVH6* by facilitating H3K9ac and H3K14ac to maintain thermotolerance in *Arabidopsis* (Hu et al., 2015). In maize, the down-regulation of *ZmHO-1* and *ZmGSL1* was associated with the decrease of acetylation levels in their promoter regions under heat stress, indicating that histone acetylation was involved in the regulation of genes expression in response to heat stress (Zhang et al., 2018b). Studies also show significant changes in histone acetylation and methylation, indicating their combined involvement in maize’s heat stress response (Hou et al., 2019; Yue et al., 2021). Wheat’s TaHAG1 and TaNACL interaction enhances heat tolerance (Lin et al., 2022), while *Arabidopsis*’s HD2C and SWI/SNF complex interaction suppresses heat-activated genes (Buszewicz et al., 2016). *Arabidopsis* also utilizes HD2B and HD2C with ARGONAUTE4 for heat tolerance through heterochromatin stabilization (Yang et al., 2023a). *ZmHDACs* downregulation and H3K9ac/H4K5ac upregulation in histones under heat suggests their critical role in heat stress response (Zhang et al., 2020b).

Heat stress is characterized by the adverse impact on plant growth and development when plants are exposed to high-temperature environments that surpass their optimal temperature range for normal physiological functioning. While ambient warm temperature conditions induce thermomorphogenesis, a process that shapes plant growth and development through a series of morphological adaptations. These adaptations include thermal acclimation, the development of thinner leaves, and the elongation of petioles and hypocotyls. Increasing evidence suggests that histone acetylation is also involved in thermomorphogenesis. MRG2 was shown to directly interact with the acetyltransferase HAM1/2, which is responsible for histone H4K5ac modification, thereby enhancing the transcription of thermal response genes such as *YUC8* and *SAUR19* (Zhou et al., 2024). One study showed that three HDACs (HDA9, HDA15, and HDA19) were involved in the thermomorphogenesis response of *Arabidopsis*. HDA15 was shown to be a direct repressor of plant thermal response process, while HDA9 and HDA19 promoted thermal response indirectly (Shen et al., 2019). Furthermore, HDA9 was reported to be involved in thermomorphogenesis in an auxin dependent manner (van der Woude et al., 2019).

Cold stress responses include HD2C degradation and the PWR-HOS15 complex recruiting CBF transcription factors and HATs to activate Cold Responsive (*COR*) gene transcription and freezing tolerance (Lim et al., 2020). In rice, cold stress induces H3K27ac but inhibits H3K27me3 to promote transcription of *COR* genes (Dasgupta et al., 2022). Cotton shows decreased acetyltransferase levels under cold (Truong et al., 2021). Under cold stress, the hyper-acetylation of H3K9 at the promoter and upstream region of the rice *dehydration responsive element binding protein 1b* (*OsDREB1b*) promoted chromatin remodeling and enhanced transcriptional activation (Roy et al., 2014). *Arabidopsis* recruits CBF factors to *COR* gene promoters, increasing H3 acetylation and activating *COR* genes (Pavangadkar et al., 2010). Similarly, the MaMYB4 factor in bananas represses *ω-3 MaFADs* transcription by modulating acetylation levels during cold stress (Song et al., 2019a). Overall, histone acetylation and methylation significantly impact plant responses to temperature stress, with HAT/HDAC regulating genes through interaction with temperature-related transcription factors.

### Light signaling

Light stress is a significant abiotic stress, with both excessive and insufficient light having detrimental effects. Research indicates that HATs/HDACs play a crucial role in plant responses to light signals. For instance, variations in light intensity influence Nitric Oxide (NO) levels, correlating with changes in histone acetylation such as H3ac, H3K9ac and H3K14ac, regulated by HDA6 (Ageeva-Kieferle et al., 2021). GCN5-HD1-TAF1 complex regulated light-responsive gene expression by altering the acetylation level of H3K9, H3K27 and H4K12 in *Arabidopsis* (Benhamed et al., 2006). The up-regulation of the photoreceptor gene *PHYA* (*PHYTOCHROME A*) was associated with an increased pattern of histone acetylation at the *PHYA* locus, indicating that the expression of the photoreceptors themselves seemed to be regulated by histone acetylation (Jang et al., 2011). HAF2, from the TAFII250 family, activates light-dependent gene expression, affecting red/far-red and blue light responses via histone acetylation (Lee and Seo, 2018; Servet et al., 2010). Mutants of GCN5 and other chromatin factors affecting H2B ubiquitination, H3K36 trimethylation and H2A.Z removal impair photomorphogenesis and light adaptation (Bieluszewski et al., 2022).

The HOS15-EC-HDA9 complex reduces the activity of the *GIGANTEA*, which is crucial for initiating flowering based on day length (Park et al., 2019). SNL-HDA19 represses *HY5* and *BBX22*, affecting *Arabidopsis* photomorphogenesis (Jing et al., 2021). The HY5-HDA15 complex represses cell wall and auxin signaling genes by altering the levels of histone H4 acetylation to promote photomorphogenesis in a light-dependent manner (Zhao et al., 2019). HY5 also interacted with HDA9 to repress autophagy-related genes by changing H3K9ac and H3K27ac levels, in response to light-to-dark conversion (Yang et al., 2020). The PIF3-HDA15 protein complex negatively regulated the expression of photosynthetic genes by reducing the acetylation level of target genes (Liu et al., 2013). Furthermore, HDA19 and MED25 are recruited by PIF1/PIF3 to target gene promoters, thereby playing a negative role in photochrome signalings (Guo et al., 2023b). Recent study has revealed that the direct-target genes of PIF rapidly adjusted the level of H3K9ac in response to the light signal (Gonzalez et al., 2022). This finding complements the work of Willige et al (Willige et al., 2021), which showed that PIFs also changed the level of H3K9ac responding to the change of light quality, highlighting the intricate interplay between light perception and epigenetic regulation in plants. Further research is needed to uncover more proteins involved and to understand the intricate ways they regulate plant responses to light.

### Phytohormone signaling

Phytohormones play important roles in plant growth and productivity. Recent studies suggest that several HATs/HDACs are involved in the signaling pathways of plant hormone such as ABA (Abscisic Acid), ethylene, JA (Jasmonic Acid), SA (Salicylic Acid) and BR (Brassinolide). HDA15 interacts with MYB96 to negatively regulate *RHO GTPASE OF PLANTS* (*ROP*) genes in ABA signaling via mediating the deacetylation of histone H3 and H4 (Lee and Seo, 2019). HDA15 also affects ABA responses by interacting with MAC3A/MAC3B to mediate splicing of introns (Tu et al., 2022). *Brachypodium* histone deacetylase BdHD1 regulates the expression of *BdWRKY24* by changing H3K9ac to regulate ABA and drought stress responses (Song et al., 2019b). AtHD2D interacted with CKA4 contributing to ABA response and root development (Zhang et al., 2022b). The MSI1-HDA19 complex repressed the expression of ABA-responsive genes by keeping low levels of histone H3K9ac to obtain decreased ABA sensitivity (Mehdi et al., 2016). HAT/HDAC typically regulates the ABA signaling pathway by interaction with ABA-related TFs like WRKYs and MYBs, affecting plant senescence and stress response.

In ethylene signaling, SRT1, SRT2 and ENAP1 form a complex to suppress genes by reducing H3K9ac at their promoter regions (Zhang et al., 2018a). The MdERF4-MdTPL-MdHDA19 repressor complex participates in the epigenetic regulation of fruit ripening and ethylene production by facilitating H3K9 deacetylation (Hu et al., 2022). Histone deacetylase SlHDT1 regulates the genes related to ethylene and carotenoid biosynthesis to delay tomato fruit ripening by altering total histone H3ac level (Guo, 2022b). The SlERF.F12-TPL2-HDAs protein complex regulates ripening genes in ethylene signaling by changing the level of H3K9ac and H3K27ac (Deng et al., 2022). Thus, HATs are typically involved in the ethylene signaling pathway through interaction with histone binding proteins ENAP1 and transcription factors like TPL, affecting fruit development and ripening.

The GCN5-TPL-HDA6 module maintains the homeostasis of acetylated TPL to regulate JA signaling (An et al., 2022). JA and HDA6 altered the level of H4ac and H3K27me3 to allow adaptation to environmental challenges in *Arabidopsis* (Vincent et al., 2022). Thermomorphogenesis is induced by the phytohormone auxin, HDA9 was involved in auxin accumulation and thermomorphogenesis by mediating histone deacetylation at the *YUCCA8* locus (van der Woude et al., 2019). In rice, the histone deacetylase HDA703 interacts with OsBZR1 to regulate BR signaling, growth and heading date by regulating *Ghd7* expression via histone H4 deacetylation (Wang et al., 2020). Histone acetyltransferase HAM1 interacts with molecular chaperone DNAJA2 and confers immune responses by promoting H3K9ac and H4K5ac of salicylic acid biosynthetic genes in cassava (Zhao et al., 2023).

## Conclusion and perspectives

**Figure 2:**
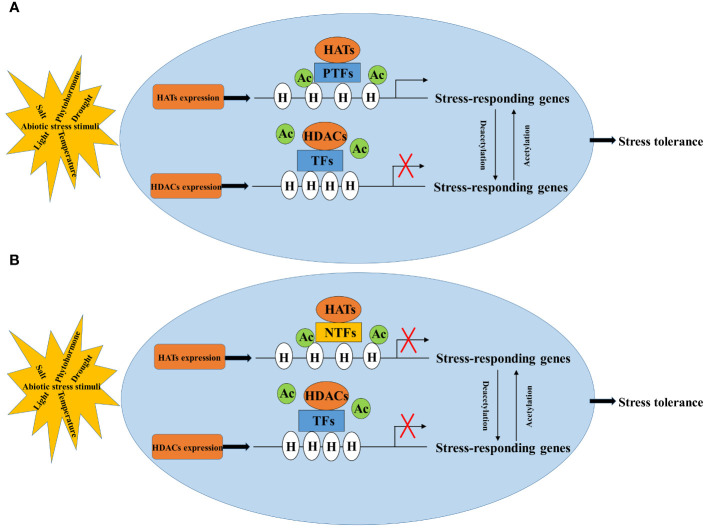
Regulatory pattern of histone acetylation in response to abiotic stress. NTFs, negative transcription factors **(B)**; PTFs, positive transcription factors **(A)**; HATs, histone acetylases; HDACs, histone deacetylases; H, histone; Ac, acetylation.

The regulation of abiotic stress in plants involves a complex process where HATs/HDACs cannot work alone. They need to cooperate with transcription factors or protein complexes to regulate the expression of stress-responsive genes (Kim et al., 2010; Chen et al., 2019; Gao et al., 2021a; Deng et al., 2022; Zhao et al., 2023). Acetylation generally makes the chromatin structure more open, allowing genes to be more easily influenced by regulatory factors like transcription factors. However, whether a gene is turned on or off depends on whether the transcription factor acts as a positive or negative regulator. Therefore, identifying and understanding how transcription factors interact with HATs/HDACs (**Figure 2**) is crucial in regulation of crop plants under abiotic stress through histone acetylation.

HATs/HDACs interact with diverse DNA-binding transcriptional factors forming multiple protein complexes to regulate the chromatin structure and the gene expression in plant responses to stresses. Identifying the transcriptional factors that interact with HATs/HDACs through yeast two-hybrid screening, *in vivo* immunoprecipitation in combination with mass spectrometry (IP-MS) or pull down in combination with mass spectrometry (Pull down-MS) is vital for mapping out the protein-protein interaction networks in the regulation of abiotic stress responses (Hou et al., 2022). To further understand how HATs are involved in plant responses to abiotic stress, it is also important to identify the transcriptional regulatory network and the genome-wide binding site of HATs regulated histone modification by using RNA-seq and ChIP-seq approaches (**Supplementary Table 3**). Techniques like ChIP-qPCR, ChIP-PCR, and Western blotting can analyze acetylation levels and binding sites on gene promoters (Micsinai et al., 2012; Zhang et al., 2022a) (**Supplementary Tables 2**, **3**). There are many other methods to detect histone acetylation besides ChIP-seq, ChIP-PCR and WB. For example, mass spectrometry is a commonly used method for acetylation modification detection. By hydrolyzing the acetylated protein into polypeptide fragments, the modification site and the quantity of acetylation on the protein can be determined by mass spectrometer analysis. Furthermore, novel histone modifications can also be identified by mass spectrometry. Due to the high sensitivity and accuracy of mass spectrometry, as well as large-scale analytical capabilities, it has been widely used in epigenetics research. CUT-TAG (Cleavage Under Targets and Tagmentation) developed on the basis of CUT-RUN (Cleavage Under Targets & Release Using Nuclease). CUT-TAG has emerged as a more user-friendly alternative compared to its predecessor, streamlining the process of identifying target genes. In the case of known modification sites, CUT-TAG can be used to study the enrichment of specific histone modification in the whole genome. DNase-seq (DNase I hypersensitive sites sequencing), MNase-seq (Micrococcal Nuclease digestion with deep sequencing) and ATAC-seq (Assay for Transposase-Accessible Chromatin with high throughput sequencing) also aimed to the study of histone acetylation. DNase-seq and MNase-seq require a large number of cells, and the enzyme digestion conditions are difficult to control. However, ATAC-seq requires a low number of cells and is highly sensitive. Compared with ChIP-seq and CUT-TAG, there is no need for specific antibodies such as transcription factors or histone modification antibody. In epigenetic studies, ATAC-seq can directly measure the degree of chromatin accessibility between two different samples. The level of histone acetylation and its modification site are usually determined by more than one method, making the results more convincing. Yeast one-hybrid (Y1H), dual-luciferase reporter system (Luc/Ren) and electrophoretic mobility shift assay (EMSA) can be used to identify the interaction relationships between the transcription factors and downstream genes in response to stress (Long et al., 2021; Wang et al., 2022).

Further research is required to investigate histone acetylation in more plants respond to abiotic stress beyond the commonly studied *Arabidopsis* and rice. It’s important to study this process in various plants to understand the regulatory differences and similarities. Since plants face multiple stresses simultaneously, involving complex responses where HATs interact with multiple TFs and protein complexes, often co-regulated with other modifications like methylation. Therefore, advanced research and methods are necessary to uncover the intricate components and regulatory networks associated with HATs.

In recent years, epigenetic studies in biology, medicine and model plants have laid an important foundation for studying histone acetylation in plants (EPIC Planning Committee, 2012). Improved analytical techniques now allow for more precise insights into its regulatory roles. This knowledge offers promising ways to enhance plant stress resistance by managing histone acetylation. This review covers advances in histone acetylation for abiotic stress management, outlines common research methods, and highlights its potential in boosting agricultural productivity through better plant adaptation to environmental changes.

## Author contributions

FW: Writing – original draft, Writing – review & editing. C-HL: Software, Writing – original draft. YL: Data curation, Software, Writing – original draft. L-FH: Software, Writing – original draft. PL: Investigation, Writing – original draft. J-XG: Investigation, Writing – original draft. NZ: Supervision, Writing – review & editing. BZ: Writing – review & editing. Y-DG: Writing – original draft, Writing – review & editing.
