# Supplementary material for: Plant responses to abiotic stress regulated by histone acetylation
Source: Front Plant Sci. 2024 Jul 16;15:1404977. doi: 10.3389/fpls.2024.1404977 (PMC11286584; doi:10.3389/fpls.2024.1404977)
Supplement: Supplementary file 1 [file DataSheet_1.docx]

**Table S1 Common HATs conserved domain.**

| conserved domain | gene family | function |
| --- | --- | --- |
| ELP3 | HAG | interaction with RNA Pol II during transcript elongation |
| HAT1 | HAG | Histone acetylation |
| Chromodomain | HAG/HAM | binding with specific acetylated histone residues |
| Bromodomain | HAF | binding with specific acetylated histone residues |
| Znf-C2H2 | HAG/HAM | protein-DNA interaction |
| Znf-C2HC | HAF | protein-DNA interaction |
| Znf-ZZ | HAC | protein–protein interactions |
| Znf-TAZ | HAC | protein–protein interactions |
| PHD | HAC | interaction with histones |
| KIX | HAC | binding with transcription factors and lysine residues |

**Table S2 Histone acetylation regulation in plants under abiotic stress.**

| Species | Abiotic stress | Histone modification | Research methods | References |
| --- | --- | --- | --- | --- |
| Rice | Drought stress | Histone acetylation | qPCR、WB | (Fang et al., 2014) |
| Maize | Salt stress | Histone acetylation | ChIP-qPCR | (Li et al., 2014) |
| Rice | Salt stress | Histone deacetylation | ChIP-seq、ChIP-qPCR | (Cheng et al., 2018) |
| *Arabidopsis* | Salt stress | Histone acetylation | ChIP-qPCR | (Zheng et al., 2019) |
| Millet | Drought, salt stress | Histone acetylation | Transcriptome data | (Xing et al., 2022) |
| Rice | Cold stress | Histone acetylation | ChIP-seq、ChIP-qPCR | (Dasgupta et al., 2022) |
| *Arabidopsis* | Cold stress | Histone acetylation | ChIP-qPCR | (Lim et al., 2020) |
| Apple | Drought stress | Histone acetylation | ATAC-seq | (Wang et al., 2022a) |
| *Arabidopsis* | Heat stress | Histone acetylation | ATAC-seq | (Kim et al., 2020) |
| Cotton | Cold stress | Histone acetylation | CUT-TAG | (Wang et al., 2024) |

**Table S3 Techniques for detecting histone acetylation.**

| Techniques | Techniques principle | Advantage | Disadvantages |
| --- | --- | --- | --- |
| ChIP-qPCR | Antibody enrichment | The multiple of ChIP enrichment can be calculated | Sample purity required high |
| ChIP-seq | Antibody enrichment | Large amount of information | Sample quantity large; Poor repeatability; Low signal; High background |
| ChIP-PCR | Antibody enrichment | The location of gene promoter acetylation can be determined | Sample purity required high; The level of histone acetylation cannot be compared |
| DNase-seq | The DNase cutting | Information about open chromatin can be obtained | Large cell volume; the conditions of enzyme digestion are difficult to control |
| MNase-seq | Micrococcal nuclease cutting | Information about open chromatin can be obtained | Large cell volume; the conditions of enzyme digestion are difficult to control |
| ATAC-seq | The Tn5 enzyme cutting | Simple operation; Good repeatability; Less cell volume; Single cell sequencing is possible | Large DNA fragments cannot be enriched by PCR; Tn5 enzyme activity needs to be optimized |
| CUT-TAG | Antibody enrichment | Simplify the steps of building a library | The sample type is the cell line; Cells tend to clump |
| CUT-RUN | Antibody enrichment | Low cell count; Low background; High resolution | The sample type is the cell line; Cells tend to clump |

**References**

Fang, H., Liu, X., Thorn, G., Duan, J., Tian, L. (2014). Expression analysis of histone acetyltransferases in rice under drought stress. *Biochemical and biophysical research communications*. 443, 400–405. https://doi.org/10.1016/j.bbrc.2013.11.102

Li, H., Yan, S., Zhao, L., Tan, J., Zhang, Q., Gao, F., et al. (2014). Histone acetylation associated up-regulation of the cell wall related genes is involved in salt stress induced maize root swelling. *BMC plant biology*. 14, 105. https://doi.org/10.1186/1471-2229-14-105

Cheng, X., Zhang, S., Tao, W., Zhang, X., Liu, J., Sun, J., et al. (2018). INDETERMINATE SPIKELET1 Recruits Histone Deacetylase and a Transcriptional Repression Complex to Regulate Rice Salt Tolerance. *Plant physiology*. 178, 824–837. https://doi.org/10.1104/pp.18.00324

Zheng, M., Liu, X., Lin, J., Liu, X., Wang, Z., Xin, M., et al. (2019). Histone acetyltransferase GCN5 contributes to cell wall integrity and salt stress tolerance by altering the expression of cellulose synthesis genes. *The Plant journal*. 97, 587–602. https://doi.org/10.1111/tpj.14144

Xing, G., Jin, M., Qu, R., Zhang, J., Han, Y., Han, Y., et al. (2022). Genome-wide investigation of histone acetyltransferase gene family and its responses to biotic and abiotic stress in foxtail millet (Setaria italica [L.] P. Beauv). *BMC plant biology*. 22, 292. https://doi.org/10.1186/s12870-022-03676-9

Dasgupta, P., Prasad, P., Bag, S. K., Chaudhuri, S. (2022). Dynamicity of histone H3K27ac and H3K27me3 modifications regulate the cold-responsive gene expression in Oryza sativa L. ssp. indica. *Genomics*. 114, 110433. https://doi.org/10.1016/j.ygeno.2022.110433

Lim, C. J., Park, J., Shen, M., Park, H. J., Cheong, M. S., Park, K. S., et al. (2020). The Histone-Modifying Complex PWR/HOS15/HD2C Epigenetically Regulates Cold Tolerance. *Plant physiology*. 184, 1097–1111. https://doi.org/10.1104/pp.20.00439

Wang, S., He, J., Deng, M., Wang, C., Wang, R., Yan, J., et al. (2022a). Integrating ATAC-seq and RNA-seq Reveals the Dynamics of Chromatin Accessibility and Gene Expression in Apple Response to Drought. *International journal of molecular sciences*. 23, 11191. https://doi.org/10.3390/ijms231911191

Kim, S., Piquerez, S. J. M., Ramirez-Prado, J. S., Mastorakis, E., Veluchamy, A., Latrasse, D., et al. (2020). GCN5 modulates salicylic acid homeostasis by regulating H3K14ac levels at the 5' and 3' ends of its target genes. *Nucleic acids research*. 48, 5953–5966. https://doi.org/10.1093/nar/gkaa369

Wang, J., Liang, Y., Gong, Z., Zheng, J., Li, Z., Zhou, G., et al. (2024). Genomic and epigenomic insights into the mechanism of cold response in upland cotton (Gossypium hirsutum). *Plant physiology and biochemistry*. 206, 108206. https://doi.org/10.1016/j.plaphy.2023.108206
